# Frailty and functional decline after emergency abdominal surgery in the elderly: a prospective cohort study

**DOI:** 10.1186/s13017-019-0280-z

**Published:** 2019-12-30

**Authors:** Hwee Leong Tan, Shermain Theng Xin Chia, Nivedita Vikas Nadkarni, Shin Yuh Ang, Dennis Chuen Chai Seow, Ting Hway Wong

**Affiliations:** 1 Department of General Surgery, Singapore General Hospital, 20 College Road, Academia Level 5, Singapore, 169856 Singapore; 2 SingHealth Internal Medicine Residency, Singapore General Hospital, 20 College Road, Academia Level 3, Singapore, 169856 Singapore; 3 Centre for Quantitative Medicine, Duke-NUS Graduate Medical School, 8 College Rd, Singapore, 169857 Singapore; 4 Nursing Division, Nursing Quality, Research & Transformation, Singapore General Hospital, Outram Road, Singapore, 169608 Singapore; 5 Department of Geriatric Medicine, Singapore General Hospital, 20 College Road, Academia Level 3, Singapore, 169856 Singapore; 6 Duke-NUS Medical School, 8 College Rd, Singapore, 169857 Singapore

**Keywords:** Emergency surgery, Abdominal surgery, Elderly, Frailty, Functional independence, Functional decline

## Abstract

**Background:**

Frailty has been associated with an increased risk of adverse postoperative outcomes in elderly patients. We examined the impact of preoperative frailty on loss of functional independence following emergency abdominal surgery in the elderly.

**Methods:**

This prospective cohort study was performed at a tertiary hospital, enrolling patients 65 years of age and above who underwent emergency abdominal surgery from June 2016 to February 2018. Premorbid variables, perioperative characteristics and outcomes were collected. Two frailty measures were compared in this study—the Modified Fried’s Frailty Criteria (mFFC) and Modified Frailty Index-11 (mFI-11). Patients were followed-up for 1 year.

**Results:**

A total of 109 patients were prospectively recruited. At baseline, 101 (92.7%) were functionally independent, of whom seven (6.9%) had loss of independence at 1 year; 28 (25.7%) and 81 (74.3%) patients were frail and non-frail (by mFFC) respectively. On univariate analysis, age, Charlson Comorbidity Index and frailty (mFFC) (univariate OR 13.00, 95% CI 2.21–76.63, *p* < 0.01) were significantly associated with loss of functional independence at 1 year. However, frailty, as assessed by mFI-11, showed a weaker correlation than mFFC (univariate OR 4.42, 95% CI 0.84–23.12, *p* = 0.06). On multivariable analysis, only premorbid frailty (by mFFC) remained statistically significant (OR 15.63, 95% CI 2.12–111.11, *p* < 0.01).

**Conclusions:**

The mFFC is useful for frailty screening amongst elderly patients undergoing emergency abdominal surgery and is a predictor for loss of functional independence at 1 year. Including the risk of loss of functional independence in perioperative discussions with patients and caregivers is important for patient-centric emergency surgical care. Early recognition of this at-risk group could help with discharge planning and priority for post-discharge support should be considered.

## Background

The number of surgical procedures in the elderly population has increased in the past few decades [1–4]. In the USA, half of all operations are performed in patients over 65 years of age [3], with the ageing population estimated to increase surgical workload by almost 50% by 2020 [4]. While older patients undergoing surgery have higher mortality and morbidity risks [5, 6], chronological age alone may be a poor predictor [7].

Frailty, a decrease in physiological reserve, is associated with multisystem impairments [8, 9], and appears superior to chronological age in predicting outcomes in elderly patients [10, 11]. There are more than 20 commonly used frailty instruments [12, 13], with Fried’s Frailty Criteria (FFC) [14] and Modified Frailty Index-11 (mFI-11) [15] being commonly used in surgical patients [16, 17]. Other scales were not examined in this present study to avoid participant fatigue in this group which were generally recruited post-surgery.

Frailty has been associated with increased risk of postoperative complications, longer length of stay, discharge to step-down care, loss of functional independence and higher readmission rates in post-surgical patients [3, 11, 18]. However, due to the difficulty in administering frailty scores in emergency surgical patients, these studies were mostly conducted in elective surgical patients [3, 11, 18]. Studies of frailty in emergency surgical patients are limited [19–23]. The objective of our study was to examine the impact of preoperative frailty on loss of functional independence following emergency abdominal surgery in the elderly.

## Methods

### Study design

This prospective cohort study was performed at the Singapore General Hospital, the largest tertiary hospital in Singapore. We prospectively enrolled patients 65 years of age and above who underwent emergency abdominal surgery (including diagnostic laparoscopies and emergency abdominal wall hernia repairs) from June 2016 to February 2018. Written informed consent was obtained prior to enrolment in the study. Vascular, gynaecological and transplant surgeries and emergency operations for complications of elective surgery were excluded. For patients who were cognitively impaired, their next-of-kin were recruited into the caregiver arm of the study and a surrogate questionnaire was used. Patients who were not expected to survive the index admission were excluded. Patients whose cognitive state precluded informed consent, and who had no next-of-kin to consent to the caregiver arm of the study, were excluded. The SingHealth Centralised Institutional Review Board approved the study (2016/2338).

### Study protocol and definitions

All patients were approached postoperatively in the general ward. Upon recruitment, premorbid characteristics including demographic information, medical comorbidities (scored using the Charlson Comorbidity Index [CCI]), nutritional status (assessed using the Malnutrition Universal Screening Tool [MUST]), cognitive function (assessed using the Mini Mental State Examination [MMSE]), functional independence (by Modified Barthel’s Index) and frailty measures (Modified Fried’s Frailty Criteria and Modified Frailty Index-11) were assessed. Perioperative characteristics (diagnosis, type of surgery and surgical approach) and outcomes (postoperative intensive care unit admission, morbidity (defined and graded using the Clavien-Dindo classification), length of hospitalisation, reoperation and 30-day unplanned readmission) were collected. Patients were followed-up for 1 year, with reassessment of functional independence and unplanned readmission at follow-up (30 days, 90 days, 6 months and 1 year). The primary outcome was loss of functional independence at 1 year and its predictors.

### Frailty measures

#### Modified Fried’s Frailty Criteria (mFFC)

The primary measure of frailty in the study was mFFC, shown in a preliminary analysis of this cohort to have a stronger association with poor outcomes [24]. The mFFC is a multi-dimensional screening tool comprising the five domains of grip strength, exhaustion, low physical activity, weight loss and slowness [14]. In this study, one of the five domains in Fried’s Frailty Criteria was modified for the emergency surgical population (time up and go, replaced by question on pre-morbid speed of crossing the road) [3]. Grip strength was measured using a Jamar hand dynamometer, compared against normative data adjusted for age and gender. Participants met the “weak grip strength” criterion if grip strength was below the 20th percentile [25]. Exhaustion was assessed using the two questions from the Centre for Epidemiologic Studies Depression (CES-D) scale: “I felt that everything that I did was an effort” and “I could not get going”. The criterion was met when participants answered “most of the time” to at least one question. Low physical activity was measured using the Global Physical Activity Questionnaire (GPAQ) developed by the World Health Organization (WHO) [26]. The criterion was met when participants failed to meet the recommended Total Physical Activity Metabolic Equivalent minutes per week of 600. The criterion for weight loss was met if participants suffered an unintentional loss of 5 kg or more in the past year. Slowness was assessed by the patient’s premorbid ability to reach the other side of the road before the light changes at a traffic light junction [16]. If the participant chose any reply other than “yes, without any difficulty”, the criterion was met. This was to replace the “time up and go” test that would not have been easy to obtain, and not reflective of premorbid ability, in emergency general surgery patients. Frailty status was then defined according to the total number of positive frailty criterion met (> 3, Frail; 1–2, Pre-frail; 0, Robust) [14]. “Pre-frail” and “Robust” patients were taken collectively as “Non-frail” for the purposes of dichotomising premorbid frailty status by mFFC.

#### Modified Frailty Index-11 (mFI-11)

The alternative frailty measure used was the mFI-11, which is an 11-point scoring system comprising 11 possible comorbidities and/or deficits as follows: diabetes mellitus; congestive cardiac failure; hypertension requiring medication; history of either transient ischemic attack or cerebrovascular accident; functional status that is non-independent; history of myocardial infarction; history of peripheral vascular disease or rest pain; history of cerebrovascular accident with neurological deficit; history of either chronic obstructive pulmonary disease or pneumonia; history of either prior percutaneous coronary intervention, previous coronary surgery or history of angina; history of impaired sensorium [15]. Each mFI-11 component was assigned one point, for a maximum of 11 points, and the frailty status was defined according to the total score (> 3, Frail; 1–2, Pre-frail; 0, Absence of frailty) [15]. “Pre-frail” and “Absence of frailty” patients were taken collectively as “Non-frail” for the purposes of dichotomising premorbid frailty status by mFI-11.

### Statistical analysis

Statistical analysis was performed using SPSS Statistics Version 19.0 (Armonk NY: IBM Corp). Continuous variables were summarised by median (interquartile range, IQR) and categorical variables by frequency (%). Continuous and categorical variables were analysed using the Mann-Whitney *U* test and chi-square test or Fischer’s exact test respectively, with a statistical significance level of 0.05 used. In the sub-group of patients who were functionally independent at baseline (Modified Barthel’s Index ≥80), univariate and multivariable logistic regression (on variables with *p* < 0.10 on univariate analysis) were performed to identify factors associated with loss of functional independence at 1 year (Modified Barthel’s Index < 80).

## Results

### Baseline and perioperative characteristics

A total of 109 patients fulfilling the inclusion criteria were prospectively recruited. The baseline demographic and perioperative characteristics are summarised in Table 1. For frailty, by the mFFC, there were 28 (25.7%) and 81 (74.3%) patients who were frail and non-frail (73 pre-frail and 8 robust) respectively prior to admission. Using mFI-11, there were 22 (20.2%) and 87 (79.8%) patients who were frail and non-frail (68 pre-frail and 19 absence of frailty) respectively prior to admission. There were 73 (66.9%) patients who completed 1-year follow-up.

**Table 1:** Baseline demographics, perioperative characteristics and unplanned readmission rates of frail vs non-frail patients (by Modified Fried’s Frailty Criteria)

Characteristic	All (*n* = 109)	Frail (*n* = 28)	Non-frail (*n* = 81)	*P* value
Median age (IQR), years	74 (69–78)	71 (68–77)	74 (69–79)	0.45
Male gender, *n* (%)	58 (53.2)	15 (53.6)	43 (53.1)	0.97
Ethnicity, *n* (%)				
Chinese	96 (88.1)	24 (85.7)	72 (88.9)	0.12
Malay	8 (7.3)	4 (14.3)	4 (4.9)	
Indian	5 (4.6)	0 (0.0)	5 (6.2)	
Others				
Baseline CCI, *n* (%)				
CCI ≤ 1	52 (47.7)	8 (28.6)	44 (54.3)	*0.02*
CCI > 1	57 (52.3)	20 (71.4)	37 (45.7)	
Baseline MUST score, *n* (%)				
MUST ≤ 1	43 (39.4)	7 (25.0)	36 (44.4)	0.07
MUST > 1	66 (60.6)	21 (75.0)	45 (55.6)	
Baseline cognitive impairment (MMSE score < 24)	35 (32.1)	12 (42.9)	23 (28.4)	0.16
Baseline functionally independent, *n* (%)	101 (92.7)	22 (78.6)	79 (97.5)	*0.003*
Diagnosis, *n* (%)				
Appendicitis	18 (16.5)	1 (3.6)	17 (21.0)	0.39
Cholecystitis	20 (18.3)	5 (17.9)	15 (18.5)	
Perforated viscus	16 (14.7)	5 (17.9)	11 (13.6)	
Small bowel obstruction	22 (20.2)	8 (28.6)	14 (17.3)	
Large bowel obstruction	10 (9.2)	2 (7.1)	8 (9.9)	
Ischemic bowel	7 (6.4)	3 (10.7)	4 (4.9)	
Complicated herniae	14 (12.8)	4 (14.3)	10 (12.3)	
Others	2 (1.8)	0 (0.0)	2 (2.5)	
Laparotomy, *n* (%)	68 (62.4)	20 (71.4)	48 (59.3)	0.25
Postoperative morbidity, *n* (%)	38 (34.9)	17 (60.7)	21 (25.9)	*0.001*
Major postoperative morbidity (Clavien Dindo score ≥ 3), *n* (%)	7 (6.4)	3 (10.7)	4 (4.9)	0.37
Postoperative intensive care unit admission, *n* (%)	17 (15.6)	7 (25.0)	10 (12.3)	0.11
Reoperation, *n* (%)	7 (6.4)	1 (3.6)	6 (7.4)	0.68
Median length of stay (IQR), days	12 (8-19)	17 (11-25)	10 (7-17)	*0.005*
30-day unplanned readmission	29 (26.6)	11 (39.3)	18 (22.2)	0.08
1-year unplanned readmission	49 (45.0)	18 (64.3)	31 (38.3)	*0.02*

*IQR* Interquartile range, *CCI* Charlson Comorbidity Index, *MUST* Malnutrition Universal Screening Tool, *MMSE* Mini Mental State Examination

Frailty (by mFFC) was significantly associated with higher rates of postoperative morbidity (60.7% vs 25.9%, *p* = 0.001), longer median length of stay (17 vs 10 days, *p* = 0.005) and higher rates of unplanned readmissions at 1 year (64.3% vs 38.3%, *p* = 0.02) (Table 1).

### Functional independence

At baseline, 101 (92.7%) were functionally independent (mBI ≥ 80/100), of whom 69 (68.3%) completed planned follow-up at 1 year. Of these patients, seven (6.9%) had loss of independence at 1 year (Modified Barthel’s Index < 80/100) (Table 1).

On univariate analysis, age, Charlson Comorbidity Index and frailty (mFFC) (univariate OR 13.00, 95% CI 2.21–76.63, *p* < 0.01) were significantly associated with loss of functional independence at 1 year. However, frailty, as assessed by mFI-11, showed a weaker correlation than mFFC (univariate OR 4.42, 95% CI 0.84–23.12, *p* = 0.06) (Table 2). On multivariable analysis, only premorbid frailty (by mFFC) remained statistically significant (OR 15.63, 95% CI 2.12–111.11, *p* < 0.01, Table 2).

**Table 2:** Factors associated with loss of functional independence at 1 year

Factor	Univariate OR	95% CI	*P* value	Multivariable OR	95% CI	*P* value
Frail (mFFC score ≥ 3)	13.00	2.21–76.63	*0.001*	15.63	2.12-111.11	*0.007*
Frail (mFI–11 score > ≥3	4.42	0.84–23.12	0.06			
Age	1.10	0.99–1.23	0.09	1.14	1.00–1.29	0.06
Male gender	0.58	0.12–2.81	0.49			
CCI > 1	6.00	0.68–52.80	0.07	6.29	0.41–100.00	0.19
MUST score > 1	1.69	0.30–9.40	0.55			
MMSE < 24	1.83	0.37–9.03	0.45			
Laparotomy	1.69	0.30–9.40	0.55			
Postoperative morbidity	3.26	0.66–16.05	0.13			

*OR* Odds ratio, *mFFC* Modified Fried’s Frailty Criteria, *mFI–11* Modified Frailty Index–11, *CCI* Charlson Comorbidity Index, *MUST* Malnutrition Universal Screening Tool, *MMSE* Mini Mental State Examination

**Fig. 1:**
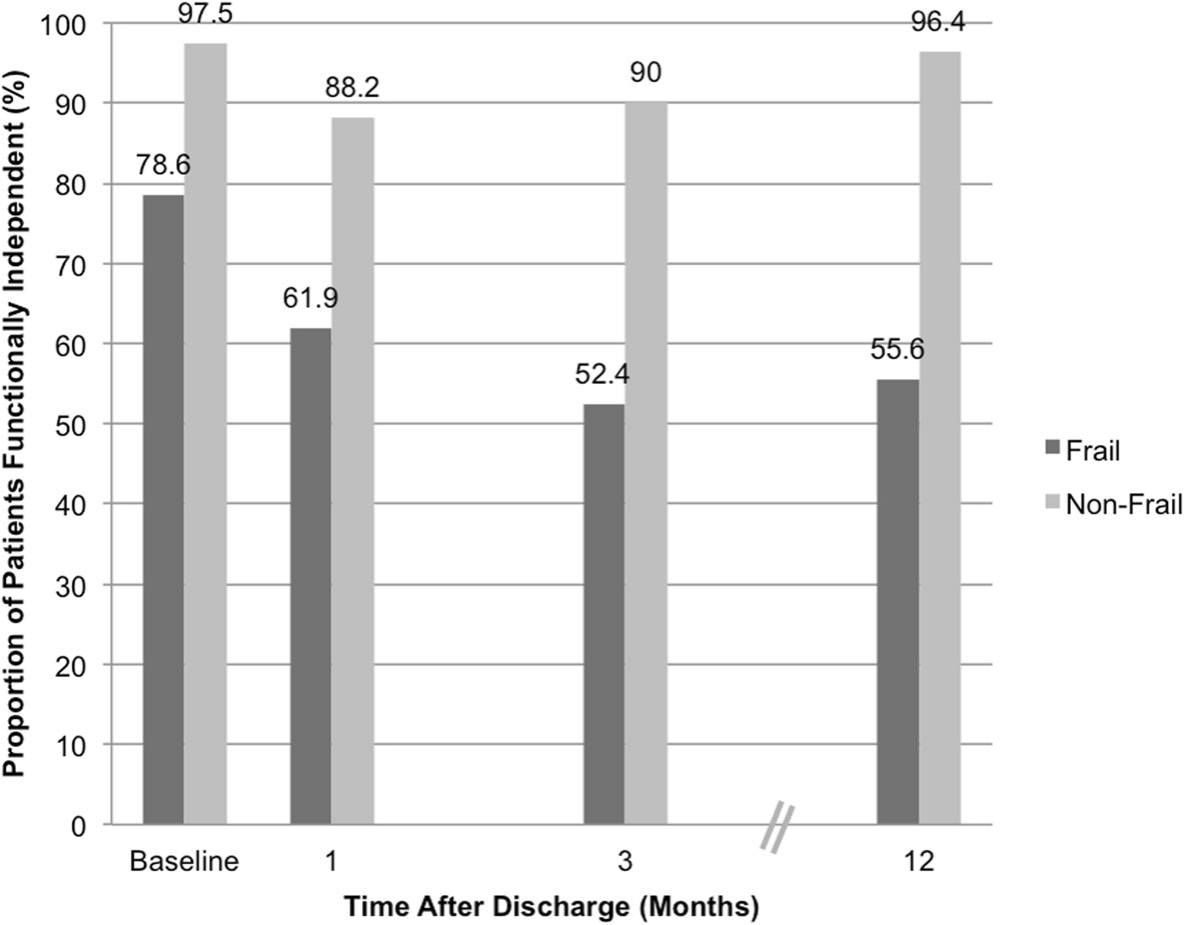
Proportion of functionally independent patients at time of discharge

When examining the temporal trend of functional independence of the entire cohort, the baseline frail (mFFC) group had a consistently lower proportion of patients who were functionally independent (Fig. 1). Most of the non-frail (mFFC) group functionally independent at baseline remained independent at 1 year (97.5% and 96.4% respectively), but the frail (mFFC) group saw a marked drop in functional independence (78.6% at baseline to 55.6% at 1 year).

## Discussion

Loss of functional independence has gained increasing recognition as a high-priority patient-centred outcome with long-term implications on quality of life and healthcare costs [27, 28]. Apart from the direct consequence of an increased need for post-discharge or institutional care, loss of functional independence has also been established as an independent predictor for recurrent readmissions and post-discharge deaths [27]. Our findings show that, even for functionally independent older patients undergoing emergency general surgery, loss of functional independence is a significant risk in frail patients, many of whom will not screen positive for frailty if the comorbidity-dominant scoring systems such as the mFI-11 are used, as opposed to phenotypic measures such as the mFFC. It is important to look beyond diagnostic labels of medical comorbidities and examine multi-dimensional phenotypic manifestations of frailty (physical strength, speed, activity, nutritional status and fatigue), which appear to be a better estimate of the physiologic reserve required to withstand perioperative stress, particularly in an emergency setting. Including the risk of loss of functional independence in perioperative discussions with patients and caregivers is important for patient-centric emergency surgical care.

Many studies have shown strong associations between frailty and poorer healthcare-related outcomes in both surgical and non-surgical patients [3, 9–11, 17, 18]. The majority of the literature on frailty in surgical patients have however been derived from elective surgical cohorts and focused on shorter-term perioperative outcomes [11, 17, 18]. In this respect, this study found preoperative frailty to be associated with higher postoperative morbidity rates and longer median hospital stay, concordant with the limited number of existing studies with elderly emergency surgical cohorts [19–23, 29]. Frailty was also found to be positively correlated to a higher baseline comorbidity burden (by Charlson Comorbidity Index) in our study, which increases the susceptibility of frail patients to perioperative adverse events and poorer postoperative healing [14].

Looking beyond the immediate postoperative period, this study found preoperative frailty to be predictive of poorer long-term functional outcomes, even for patients who were functionally independent at baseline. Amongst the patients who were functionally independent at baseline, we found higher odds of losing functional independence at 1 year amongst those with preoperative frailty. Donald et al. recently reported a similar association following elective vascular surgery alongside higher rates of discharge to a non-home location and 30-day mortality [30]. While both frail and non-frail patients saw an initial decrease in proportion of functional independence at the 30-day follow-up, the non-frail group demonstrated an upturn thereafter to reach a similar proportion at the 1-year mark, in contradistinction to the frail group which demonstrated a persistently lowered proportion of functional independence on follow-up. Lawrence et al. mapped the temporal course of functional recovery in a cohort of elderly patients undergoing elective abdominal surgery and reported poorer preoperative physical status as an independent predictor for protracted functional recovery [27]. This highlights the adverse impact of preoperative frailty extending well beyond the immediate postoperative period. Screening for baseline frailty in emergency general surgery patients would help identify elderly patients who may benefit from more intensive and prolonged postoperative rehabilitative care to ensure that surgery not only prolongs life but also preserves as much quality of life as possible [21, 27]. This is exemplified by a systematic review by Shepperd et al. which reported that a personalised and detailed approach to discharge planning was significantly associated with decreased length of stay and readmission rates [31]. We are examining the impact of incorporating these frailty scores on decision-making at our institution, and hope that our study findings would encourage adoption of frailty screening in the wider surgical community.

This study has several limitations, including the relatively small sample size, lack of standardisation of postoperative care protocols across managing surgeons, and a lost-to-follow-up rate of 33.1%. It would be difficult to extrapolate our study findings to cognitively impaired patients who do not have caregivers and potentially high-risk patients, as we could not recruit them into the study. On the other hand, the study’s prospective cohort design facilitated standardised and complete data collection which is of particular value in the use of rigorous measures of various pre- and postoperative variables such as frailty and functional independence, and featured a 1-year follow-up period to examine the temporal trends of longer-term functional independence, a dimension not widely available in existing literature. To the best of our knowledge, this represents the first study to examine the relationship between frailty and functional independence outcomes in the elderly after emergency abdominal surgery. This study informs potential future research examining patient-centred decision-making in emergency surgery situations, as well as the potential benefit of holistic rehabilitative intervention programmes amongst frail elderly patients undergoing emergency abdominal surgery.

## Conclusion and implications

The mFFC is a useful tool for frailty screening amongst elderly patients undergoing emergency abdominal surgery as a predictor for loss of functional independence at 1 year. Including the risk of loss of functional independence in perioperative discussions with patients and caregivers is important for patient-centric emergency surgical care. Early recognition of this at-risk patient subgroup with dedicated and detailed postoperative rehabilitation and discharge planning should be considered to help mitigate enduring functional impairments in the long-term.

## Data Availability

The datasets generated and analysed during the current study are not publicly available due to institutional patient confidentiality policies, but are available from the corresponding author on reasonable request.
